# Epigenetic Regulation and Neurodevelopmental Disorders: From MeCP2 to the TCF20/PHF14 Complex

**DOI:** 10.3390/genes15121653

**Published:** 2024-12-23

**Authors:** Gaea Dominguez, Yongji Wu, Jian Zhou

**Affiliations:** 1 Department of Human Genetics, Emory University School of Medicine, Atlanta, GA 30322, USA; gaea.charlize.dominguez@emory.edu (G.D.); yongji.wu@emory.edu (Y.W.); 2 Department of Pediatrics, Emory University School of Medicine, Atlanta, GA 30322, USA

**Keywords:** neurodevelopmental disorders, chromatin regulators, transcription, neuronal activity, MeCP2, RAI1, TCF20, PHF14, HMG20A, Rett syndrome

## Abstract

Background: Neurodevelopmental disorders (NDDs) affect approximately 15% of children and adolescents worldwide. This group of disorders is often polygenic with varying risk factors, with many associated genes converging on shared molecular pathways, including chromatin regulation and transcriptional control. Understanding how NDD-associated chromatin regulators and protein complexes orchestrate these regulatory pathways is crucial for elucidating NDD pathogenesis and developing targeted therapeutic strategies. Recently, the TCF20/PHF14 chromatin complex was identified in the mammalian brain, expanding the list of chromatin regulatory remodelers implicated in NDDs. This complex—which includes MeCP2, RAI1, TCF20, PHF14, and HMG20A—plays a vital role in epigenetic and transcriptional regulation. Methods: We review and summarize current research and clinical reports pertaining to the different components of the MeCP2-interacting TCF20/PHF14 complex. We examine the NDDs associated with the TCF20/PHF14 complex, explore the molecular and neuronal functions of its components, and discuss emerging therapeutic strategies targeting this complex to mitigate symptoms, with broader applicability to other NDDs. Results: Mutations in the genes encoding the components of the MeCP2-interacting TCF20/PHF14 complex have been linked to various NDDs, underscoring its critical contribution to brain development and NDD pathogenesis. Conclusions: The MeCP2-interacting TCF20/PHF14 complex and its associated NDDs could serve as a model system to provide insight into the interplay between epigenetic regulation and NDD pathogenesis.

## 1. Introduction

Neurodevelopmental disorders (NDDs) describe a broad range of neurological conditions that affect the proper development and function of the brain and nervous system. NDDs are highly prevalent, affecting approximately 15% of children and adolescents worldwide [1]. These disorders typically manifest in early childhood, leading to impairment of a variety of functions, including cognitive, motor skills, communication, and behavior [2,3,4]. Examples of NDDs include attention-deficit/hyperactivity disorder (ADHD), autism spectrum disorder (ASD), intellectual disability (ID), schizophrenia (SCZ), and movement disorders, among others. The genetic causes of NDDs are complex and often polygenic, making it challenging to delineate a genotype–phenotype correlation. Genome-wide association studies (GWAS) have identified numerous risk loci for NDDs, many of which encode epigenetic regulators that control gene expression through mechanisms such as DNA methylation, histone modifications, chromatin remodeling, and transcriptional regulation [5,6,7]. Notably, many NDD-associated genes are involved in overlapping molecular pathways, particularly those related to chromatin regulation and transcriptional control [8,9,10,11]. The convergence of genetic variants in chromatin regulators raises fundamental questions about the specific protein complexes that are implicated in NDDs.

Many NDD-associated chromatin regulators function in the same protein complex and converge on shared downstream pathways [5,12]. For example, mutations affecting components of the ATP-dependent BRG1/BRM-associated factor (BAF) chromatin-remodeling complex have been linked to NDDs such as Coffin–Siris syndrome [13,14], various ID-related conditions [7,15,16,17,18], and ASDs [19,20]. The nucleosome remodeling and deacetylase (NuRD) complex, which includes proteins such as histone deacetylase 1 (HDAC1) and 2 (HDAC2), is another ATP-dependent chromatin remodeler complex whose impaired function leads to developmental delay, IDs, and ASDs, among other disorders [21,22,23,24]. Other chromatin regulators implicated in NDDs are the Polycomb repressive complexes 1 (PRC1) and 2 (PRC2). PRC1 mediates monoubiquitylation of histone H2A, while PRC2 mediates histone H3 lysine 27 (H3K27) methylation [25]. Mutations affecting these histone-modifying regulators are implicated in various IDs, ASDs, and neurological syndromes like Gabriele de Vries Syndrome [26,27,28,29,30]. The cohesin complex plays a crucial role in chromatid cohesion, gene expression regulation, and DNA repair. Pathogenic variants in cohesin pathway genes—such as NIPBL, SMC1A, SMC3, RAD21, and HDAC8—cause Cornelia de Lange syndrome, a severe genetic disorder characterized by multisystem malformations [31,32,33]. These findings shed light on how disruptions in these complexes could result in transcriptional dysregulation leading to developmental abnormalities, underscoring the critical role of chromatin regulators in maintaining the epigenetic environment essential for healthy neurodevelopment.

Rett syndrome (RTT) is one of the most frequent NDDs that is caused by loss-of-function (LoF) variants in the X-linked gene methyl-CpG–binding protein 2 (MECP2) [34,35,36]. *MECP2* encodes an epigenetic regulator that binds broadly to methylated DNA across the genome [37]. Transcriptomic analyses in mice lacking *Mecp2* reveal widespread changes in the expression of thousands of genes, highlighting the broad impact of MeCP2 dysfunction on gene regulation [38,39,40,41]. Since MeCP2 lacks a transactivation or catalytic domain, it is believed to function as a bridge between methylated DNA and other regulatory elements. Supporting this hypothesis, MeCP2 has been shown to interact with several chromatin-modifying complexes, including the NCoR complex, SIN3A complex, CoREST complex, and others [37,42,43,44]. Understanding the mechanism by which MeCP2 interacts with these chromatin complexes is crucial for elucidating the pathogenesis of RTT, and perhaps even other NDDs.

**Figure 1:**
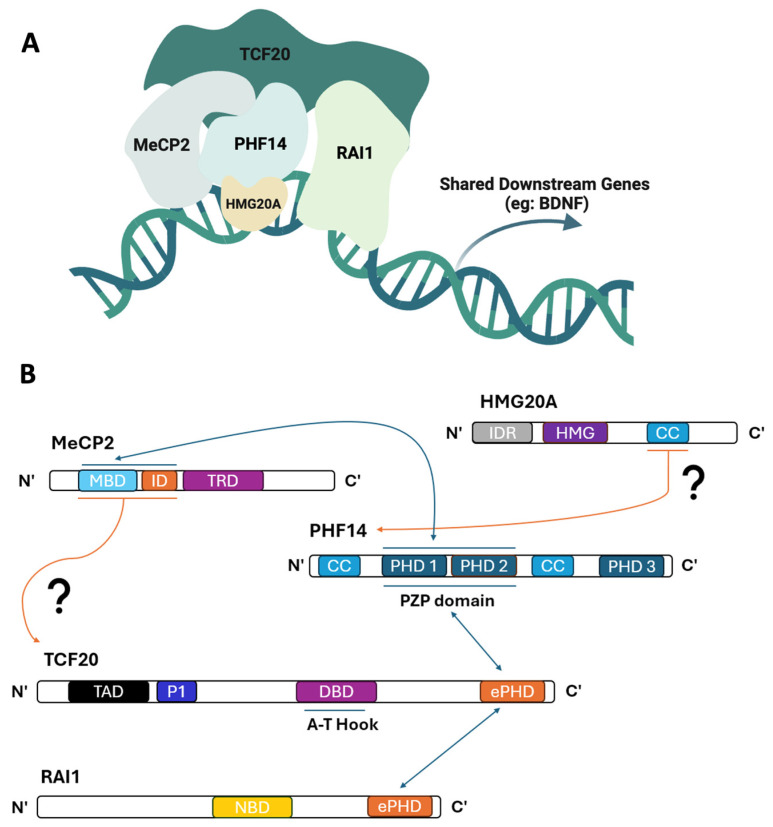
Structural features of the MeCP2-interacting TCF20/PHF14 protein complex, its components, and the known interactions between them. The MeCP2-interacting TCF20/PHF14 protein complex (**A**) and their known interaction (**B**). This figure illustrates the interactions between the components of TCF20/PHF14 protein complex based on biochemical data. The complex is hypothesized to modulate shared downstream targets as depicted in (**A**). Blue arrows denote interactions where the domains between proteins have been mapped. Orange arrows pointing from protein domains to a protein name indicate that the interaction between the protein domains has not been fully established. N’: N-protein terminus; C’: C-protein terminus; IDR: intrinsically disordered domain; HMG: high-mobility group; CC: coiled-coil domain; MBD: methyl-CpG-binding domain; ID: intervening domain; TRD: transcriptional repression domain; PHD: plant homeodomain; TAD: transactivation domain; P1: PEST1 domain; DBD: DNA-binding domain; NBD: nucleosome-binding domain.

Recently, a new chromatin regulatory complex was identified in the mammalian brain, which consists of MeCP2, TCF20, PHF14, and HMG20A (Figure 1A) [41]. Most of its components—TCF20, PHF14, and HMG20A—were also previously identified through a cell-based proteomic approach together with RAI1, another chromatin-binding transcription regulator [45,46]. Who are these protein players besides MeCP2, and what is their relationship to NDDs? RAI1 is a transcription factor linked to the neurodevelopmental condition Smith–Magenis syndrome [46,47]. TCF20 is another transcription factor whose mutations lead to ASD and TCF20-associated neurodevelopmental disorders [48,49,50]. PHF14 is a histone-binding protein implicated in a cerebellum-related developmental disorder called Dandy–Walker syndrome [51]. Recently, variants in PHF14 have been identified in individuals with NDDs and ASDs [41]. HMG20A is a component of a co-repressor complex that modulates the dynamics of neuronal differentiation [52,53]. These proteins share a common function in regulating chromatin and gene expression, but the detailed molecular roles of some of the players, such as TCF20 and PHF14, have yet to be fully determined.

Here, we review evidence from human genetics, biochemistry, developmental biology, and transcriptional studies that suggest the MeCP2-interacting TCF20/PHF14 complex plays vital roles during normal brain development and NDD pathogenesis. We begin by examining the genetic and clinical features of the NDDs associated with this complex, as summarized in Table 1, and transition to a deeper dive into their molecular and neuronal functions. Finally, we review potential therapeutic avenues that might be pursued for NDD-related treatments.

## 2. The TCF20/PHF14 Complex and Its Associated NDDs

### 2.1. MeCP2 and Rett Syndrome

Rett syndrome (RTT; MIM 312750) is a postnatal neurological disorder caused by LoF mutations in the X-linked gene methyl-CpG-binding protein 2 (*MECP2*) [36,54]. It affects approximately 1 in every 10,000 births, almost always affecting girls [55]. RTT typically presents after an initial 6 to 18 months of normal development followed by progressive neurological and developmental regression, including stereotypic handwringing, gait abnormalities, loss of speech, intellectual disability, and autistic features [36,56,57]. In addition to RTT, duplication of *MECP2*, which is often seen in boys, causes a progressive neurodevelopmental disorder called *MECP2* duplication syndrome (MDS; MIM 300260) [58]. MDS is characterized by autistic features, impaired social communication, repetitive behavior, broad psychiatric symptoms like depression, and pulmonary infections, among other symptoms [58,59,60]. Milder forms of *MECP2* mutations have been associated with a wide range of neuropsychiatric conditions, including autism [61], ID [62,63], and schizophrenia [64]. Studies have shown that mice carrying LoF mutations or duplication of *Mecp2* yield similar neurological phenotypes mimicking RTT and MDS, respectively, highlighting the utility of mouse models as tools for examining the molecular mechanisms underlying *MECP2*-related NDDs [65,66]. Interestingly, MeCP2 has also been shown to modulate metabolic homeostasis, as observed in RTT patients and *Mecp2*-deficient mouse models exhibiting cholesterol- and mitochondrial-metabolism deficiencies [67,68,69,70]. These metabolic studies extend the phenotypic spectrum of RTT beyond neurodevelopment.

*MECP2* encodes a protein that binds methylated DNA via its methyl-CpG-binding domain (MBD). MeCP2 represses transcription by recruiting the histone deacetylase 3 (HDAC3) subunit of the NCoR1/2 co-repressor complex via its transcriptional repression domain (TRD) (Figure 1B) [43,71]. RTT-associated mutations in the TRD, such as the missense variant p.R306C (Table 2), disrupt MeCP2’s binding to the NCoR1/2 complex, suggesting that disruption of this interaction plays an important role in RTT pathogenesis [43]. While missense variants like p.R306C disrupt MeCP2’s interaction with the NCoR1/2 complex, other missense variants, such as p.T158A, that affect MeCP2’s MBD domain disrupt its proper binding to methylated DNA (Table 2) [72]. Interestingly, there are missense variants—such as p.R167W and p.G185V (Table 2), which sit outside MeCP2’s MBD and TRD protein domains—identified in males with ID symptoms [73,74]. These findings suggest that *MECP2* mutations affecting males do exist, who exhibit features like neonatal encephalopathy and ID.

Not all MeCP2 mutations lead to the same phenotypic severity. In both humans and mice, mutations within the TRD that completely abolish the interaction between MeCP2 and the NCoR1/2 complex cause milder forms of the disorder compared to mutations within the MBD that disrupt MeCP2’s DNA binding [75,76,77,78,79]. These observations suggest that MeCP2 might interact with other partners besides the NCoR1/2 complex to mediate its molecular functions. The TCF20/PHF14 complex is one such partner [41].

### 2.2. RAI1 and Smith–Magenis Syndrome

Another gene implicated in neurodevelopment is retinoic acid induced 1 (*RAI1*). Haploinsufficiency or mutations of *RAI1* lead to Smith–Magenis syndrome (SMS; MIM 182290), a disorder characterized by hypotonia, intellectual disability, behavioral problems, sleep disturbances, craniofacial abnormalities, and autistic features, among other characteristics [46,47,80,81,82,83,84,85,86]. The birth prevalence of SMS is approximately 1 in 15,000 [87]. The behavioral features of SMS emerge around 18 months of age and change through adulthood [88,89]. SMS is associated with deletions of the 17p11.2 locus, which covers many genes, including *RAI1.* Despite having similar overlap in phenotype, outcome differences have been reported for SMS patients with 17p11.2 deletions compared to SMS patients with mutations—such as frameshift and missense variations, including p.P242L, p.D969D, p.S1808N, and other variants (Table 2)—that only affect *RAI1* [47,85,86,90,91,92,93,94]. Similar to *MECP2*, duplications of the 17p11.2 region encompassing the *RAI1* gene cause another NDD, termed Potocki–Lupski syndrome (PTLS; MIM 610883) [95,96,97]. Although PTLS and SMS result from mutations in the same genomic region, their clinical manifestations and behavioral profiles are distinct, with some traits appearing at opposite ends of the phenotypic spectrum [98]. These observations highlight the complexity of *RAI1*-associated disorders and the genotype–phenotype differences that contribute to their variable expressivity.

*RAI1* encodes a chromatin-binding protein that regulates multiple neurodevelopmental genes and contains protein-interacting domains that include an extended plant homeodomain (ePHD)—a type of PHD domain containing more zinc finger-like structures—and a nucleosome-binding domain (NBD) (Figure 1B) [46,99,100]. The NBD of RAI1 has been shown to interact with nucleosomes both in vitro [100] and in vivo [46], suggesting that this chromatin interaction may underlie SMS and other NDD-related phenotypes associated with *RAI1* haploinsufficiency or mutations. To further investigate RAI1’s molecular and neural functions, several *Rai1*-deficient mouse models have been created and applied to study its role in vivo. These studies mapped *Rai1* expression across developing and adult nervous system tissues and demonstrated that abnormal *Rai1* gene dosage leads to SMS-like symptoms, including obesity, sleep disturbances, and craniofacial features [90,101,102,103]. Additionally, analyses of a *Rai1* conditional allele as well as a tagged *Rai1* allele have shown that RAI1 binds to promoters of genes important for neuronal communication and circuit assembly [46]. Importantly, loss of *Rai1* in GABAergic or subcortical excitatory neurons reduced learning ability in fear conditioning, while *Rai1* loss in the subcortical excitatory neurons Sim1^+^ or SF1^+^ cells caused obesity [46]. Thus, these findings provide insight into the mechanisms of *RAI1*’s dosage-sensitive function and how disrupting that function can lead to SMS and other NDDs.

### 2.3. TCF20 and TCF20-Associated Neurodevelopmental Disorder

Along with *MECP2* and *RAI1*, transcription factor 20 (*TCF20*) is another gene implicated in NDDs, causing TCF20-associated neurodevelopmental disorder (TCF20-NDD). TCF20-NDD arises from mutations of *TCF20*, which include single nucleotide variants and structural variants such as deletions and inversions encompassing the TCF20 gene. Over 100 patients have been identified with mutations in *TCF20*, and their clinical features include developmental delay, ID, autism, ataxia, hypotonia, craniofacial dysmorphisms, and seizures [48,49,50,104,105]. Missense variations identified in some patients include p.K512E, p.P1557L, p.K1710R, and p.H1909Y (Table 2), among other nonsense mutations [48,50]. Like *MECP2* and *RAI1*, duplications of *TCF20* are associated with NDD [106], indicating that precise dosage of these transcriptional regulators is crucial for proper brain development. More recent studies indicate that variants in *TCF20* are also associated with peripheral liver fibrogenesis and immune system dysfunction, suggesting the role of this gene extends beyond the central nervous system, similar to *MECP2* [107,108].

*TCF20* encodes a transcriptional co-activator, previously known as stromelysin-1 platelet-derived growth factor-responsive element binding protein (SPBP), which enhances the activity of transcription factors [109]. TCF20 contains an AT-hook DNA-binding domain also found in other chromatin-binding proteins [110], an N-terminal transactivation domain (TAD), and a C-terminal zinc finger domain (Figure 1B) [109]. The C-terminal zinc finger domain of TCF20 represents an ePHD domain and has been shown to enhance the activity of certain transcription factors, like c-Jun and SP1, while other transcription factors like PAX6 rely on TCF20’s N-terminal TAD domain for functional interaction [109]. These biochemical findings suggest that TCF20 enhances the activity of transcription factors through its distinct protein domains, providing valuable insights into their role in regulating gene expression. However, whether these in vitro findings hold true in vivo and in patients with NDDs remains to be determined.

Interestingly, *TCF20* is homologous to *RAI1*, suggesting that both genes may have originated from a gene duplication event early in vertebrate evolution [100]. Some patients with *TCF20* mutations exhibit phenotypic features reminiscent of SMS, including craniofacial abnormalities, neurological disturbance, seizure, ataxia, abnormal gait, failure to thrive, food-seeking behaviors, and sleep disturbances [50]. Given their structural similarities [100], *TCF20* and *RAI1* may transcriptionally regulate overlapping neurodevelopment genes while each also acts upon their own specific gene targets. *Tcf20* haploinsufficient mice display autistic-like behaviors, with behavioral deficits such as reduced anxiety and impaired learning and memory that resemble RTT mouse models, implying that TCF20 and MeCP2 modulate common downstream neuronal pathways [41,111]. This overlap in symptoms implies that TCF20 and MeCP2 may modulate shared downstream neuronal pathways.

### 2.4. PHF14, HMG20A, and Their Related NDDs

Other molecular players implicated in NDDs, whose roles in neurodevelopment are not yet fully characterized, include plant homeodomain finger protein 14 (*PHF14*) and high mobility group 20A (*HMG20A*), also known as inhibitor of BRAF53 (*iBRAF*). *PHF14*, residing at the 7p21.3 locus, has been linked to Dandy–Walker malformations due to deletions or duplications of this region, leading to developmental delay, hypoplasia, and craniofacial abnormalities [51]. Additionally, *PHF14* has also been linked to ASD [112], and two patients with single nucleotide or nonsense variants of *PHF14* presented neurological features such as speech impairments, developmental delay, and ID [41]. Notably, PHF14 physically interacts with MeCP2, TCF20, RAI1, and HMG20A in a chromatin complex in the mammalian brain, highlighting the importance of this complex in mediating shared downstream pathways implicated in NDDs (Figure 1A) [41]. Among the patients identified with *PHF14* variants, the missense variant p.C322G (Table 2), which impacts the PHD domain of the protein interacting with MeCP2 (Figure 1B), abolishes PHF14’s interaction with MeCP2 and TCF20. This disruption leads to clinical features in the patient that overlap with those seen in RTT [41]. These findings indicate that PHF14 may serve as a scaffold bridge in linking proteins within this complex.

In addition to its association with NDDs, PHF14 has also been implicated in regulating the cell cycle and cell proliferation in cancer [113,114,115]. These findings suggest that PHF14 may also play a similar role in the developing central nervous system by regulating the proliferation and differentiation of neural progenitor cells.

Meanwhile, although HMG20A has not yet been linked to an NDD, it has been shown to influence neural differentiation through its HMG-domain interaction with neuronal gene promoters—such as *Synapsin* and *NeuroD2*—leading to gene activation [52,53]. Further investigation into the interaction of PHF14 and HMG20A with the TCF20/PHF14 complex could provide valuable insights into their potential links to NDDs.

**Table 1:** Example NDDs and the clinical features associated with the TCF20/PHF14 protein complex.

Gene	Chromosome Locus in Humans	Protein	Type of NDDs	Clinical Features	References
MeCP2	Xq28	Methyl-CpG Binding Protein 2	Rett Syndrome (RTT)	**Neurobehavior**: Intellectual disability; Autistic features; Loss or delay of speech; Aggression; Seizures **Motor**: Gait ataxia; Hypotonia; Tremors; Hand-wringing **Morphological**: Scoliosis; Microcephaly **Metabolic and others**: Breathing abnormalities; Sleep problems; Gastrointestinal dysfunction; Obesity; Abnormal cholesterol and mitochondria metabolism	[36,67,70,116,117,118,119,120,121,122,123,124,125,126,127,128,129,130,131,132,133]
			MECP2 Duplication Syndrome (MDS)	**Neurobehavior**: Intellectual disability; Speech delay; Autistic features; Seizures **Motor**: Hypotonia; Spasticity predominantly of the lower limbs **Morphological**: Facial dysmorphism **Metabolic and others**: Feeding issues; Gastrointestinal dysfunction; Predisposition to infections	[134,135,136,137,138,139,140,141,142,143,144,145,146,147,148]
			Autism	**Neurobehavior**: Social interaction deficits; Stereotypic and repetitive behaviors	[36,149,150,151,152,153,154,155]
			Schizophrenia	**Neurobehavior**: Speech and language problems **Motor:** Motor deficit	[64,153,156,157,158]
RAI1	17p11	Retinoic Acid Induced 1	Smith–Magenis Syndrome (SMS)	**Neurobehavior:** Intellectual disability; Speech delay; Self-injurious; Attention-seeking behaviors **Motor**: Hypotonia; Motor delay; Poor reflexes **Morphological:** Anomalies include cardiac abnormalities; Distinctive facial features; Teeth abnormalities; Skeletal malformations; Multiple congenital anomalies **Metabolic and others**: Sleep problems; Feeding issues; Obesity	[47,81,85,86,88,90,92,93,94,159,160,161,162,163,164,165,166,167,168,169,170,171]
			Potocki–Lupski Syndrome (PTLS)	**Neurobehavior:** Intellectual disability; Speech delay; Attention problems; Hyperactivity; Compulsive or impulsive behaviors; Anxiety **Motor**: Hypotonia; Motor delay **Morphological:** Facial dysmorphism **Metabolic and others**: Feeding issues; Sleep problems; Heart defect; Growth delay	[95,96,98,172,173,174,175,176,177]
			Autism	**Neurobehavior**: Social interaction deficits; Stereotypic and repetitive behaviors	[82,86,89,96,178,179,180]
			Schizophrenia	**Neurobehavior**: Speech and language problems **Motor:** Motor deficit	[178,181]
TCF20	22q13	Transcription Factor 20	TCF20-associated neurodevelopmental disorders (TCF20-NDD)	**Neurobehavior:** Intellectual disability; Speech delay, Autistic features; Aggression; Anxiety; Seizures; ADHD **Motor:** Hypotonia; Dystonia; Delayed motor development; Gait ataxia; Tremors **Morphological:** Macrocephaly; Prominent forehead; Facial dysmorphism; Scoliosis **Metabolic and others**: Sleep problems; Gastrointestinal dysfunction; Obesity; Liver fibrogenesis; Immune system dysfunction	[49,50,104,105,107,108,182]
			TCF20 duplication	**Neurobehavior:** Intellectual disability; Behavioral anomalies; Speech delay; Obsessive–compulsive disorder; Tic disorder; Oppositional defiant disorder; Anxiety; Autistic features; ADHD **Motor:** Motor delay **Morphological:** Facial dysmorphism **Metabolic and others**: Feeding issues	[50,106]
			Autism	**Neurobehavior**: Social interaction deficits; Stereotypic and repetitive behaviors	[48,104,105]
PHF14	7p21	PHD Finger Protein 14	Dandy–Walker syndrome or malformations	**Morphological**: Cystic dilatation of the fourth ventricle; Enlarged posterior fossa; Complete or partial agenesis of the cerebellar vermis; Elevated tentorium cerebelli **Metabolic and others**: Anomalies included cardiac, neurological, gastrointestinal, orthopedic, and genitourinary abnormalities; Developmental delay	[51]
			Autism Spectrum disorder (ASD)	**Neurobehavior**: Intellectual disability; Speech loss of delay; Social interaction deficits **Motor:** Motor skill regression; Gait ataxia	[41]
				**Others**: Sleep problems	
HMG20A	15q24	High Mobility Group 20A	NA	NA	NA

NA: Not applicable.

**Table 2:** Pathogenic variants affecting certain protein domains of the TCF20/PHF14 protein complex. Although a few examples of mutations are listed here, a more comprehensive list of the different types of reported mutations can be found through the indicated references.

Gene	Protein Domain	Missense Mutations	References
MeCP2	MBD	R106W G118E R133C S134C T158M/A	[73,74,75,77,183,184]
	TRD	P225L R306C R309W	
	Unknown	R167W G185V	
RAI1	ePHD	S1808N	[47,85,90,92,93,94,185]
	Unknown	P242L D969D G1070R R1147Q Q1562R	
TCF20	P1	K512E	[48,49,50]
	DBD	P1557L	
	ePHD	K1710R H1909Y	
PHF14	PHD1	C322G	[41]

MBD: methyl-CpG-binding domain; TRD: transcriptional repression domain; ePHD: extended plant homeodomain; P1: PEST domain; DBD: DNA-binding domain; PHD: plant homeodomain.

## 3. Molecular and Neuronal Function of the TCF20/PHF14 Complex

Given the significant roles of the TCF20/PHF14 complex in various NDD conditions, a fundamental question arises: What are the molecular and neuronal mechanisms by which the TCF20/PHF14 complex enacts its functions? Here, we review the literature describing the molecular and neuronal functions of the individual TCF20/PHF14 complex components in brain development.

### 3.1. Molecular Function

The TCF20/PHF14 complex is critical in regulating the expression of genes necessary for neuronal development and synaptic function, particularly in the context of neurodevelopmental and neuropsychiatric disorders like RTT and NDDs. Various studies leveraging in vitro and in vivo models have been conducted to tease apart the molecular functions of each component within this complex.

#### 3.1.1. MeCP2

MeCP2 functions primarily as an epigenetic transcription regulator, controlling the expression of hundreds to thousands of downstream genes by its recognition of distinct genomic signatures (Table 3). Through its MBD domain, MeCP2 binds to methylated CpGs (mCpGs) [186], methylated cytosines outside of the CG context (mCHs) [187], and methylated CACs (mCACs) or hydroxymethylated CACs (hmCACs) [188]. It also binds to nucleosomes with H3K27me3 repressive histone marks [189]. Through various biophysical experiments, a study also showed that MeCP2 binds canonical H2A, H2B, H3, and H4 histones (Table 3) [190]. Despite extensive efforts over the years, a distinct binding motif for MeCP2 has remained elusive. A recent study suggested MeCP2 may preferentially bind modified cytosines in cytosine–adenine (CA) dinucleotide repeats, identifying a possible new DNA binding signature [191]. However, another study indicated that MeCP2 function relies on mCAC sites, regardless of their occurrence in CA repeats throughout the genome [192], leaving the biological significance of this motif and its contribution to RTT unclear [193]. Recently, MeCP2 has also been found to bind to gene enhancers in a methylation-independent manner in the mouse cortex [194]. These efforts made to identify a distinct MeCP2 binding motif highlight MeCP2’s many biological facets in ensuring proper neurodevelopment.

Lacking a transactivation or catalytic domain, MeCP2 functions by interacting with other chromatin proteins and complexes, such as the NCoR1/2 complex, the TCF20/PHF14 complex, RNA polymerase II, and many others, to either activate or repress gene expression [41,43,195,196]. Transcriptomic analyses in mice suggest that MeCP2 regulates the expression of thousands of genes [38,39,40,41]. For instance, MeCP2 binds to the promoter region of *Bdnf* and *Gdf11*, where its improper regulation leads to altered expression levels that contribute to RTT phenotypes [197,198,199]. Beyond its role as a canonical transcriptional regulator, recent studies indicate that MeCP2 also contributes to the formation of heterochromatin condensates via liquid–liquid phase separation in mouse cells [200,201]. However, another study showed that MeCP2’s ability to bind chromatin is independent of heterochromatin phase separation. In addition, it appears that mouse cellular models are highly atypical, as MeCP2 distribution is diffuse in most mammalian species, including humans [202]. The conservation of this heterochromatin condensate mechanism across mammalian species and its implications for RTT remains to be clarified.

#### 3.1.2. RAI1

RAI1 is a multifunctional transcriptional regulator essential for both neurological and metabolic homeostasis. At the molecular level, RAI1 regulates gene expression by interacting with nucleosomes through its NBD domain, specifically recognizing the active H3K4me3 histone mark (Table 3) [46,100]. This interaction enables RAI1 to modulate transcription of its target genes, many of which are involved in neuronal signaling and cellular communication. Among these targets, RAI1 has been found to bind directly to the promoter region of *Bdnf* along with other cell-cell communication-related genes such as *Htr2c*, *Pcdh20*, and *Sema3a* [46,86,203]. Functional studies in mice have shown that haploinsufficiency of *Rai1* results in reduced *Bdnf* and *Htr2c* expression, leading to dysregulation of energy homeostasis and behavioral changes [204,205,206]. These deficits manifest in hyperphagia and obesity in *Rai1*-deficient mouse models, recapitulating the metabolic disturbances observed in patients with SMS [203].

#### 3.1.3. TCF20

TCF20 is a transcriptional regulator that plays a key role in neurodevelopment by binding to the promoters of downstream genes implicated in various neurodevelopmental processes, including neural proliferation and differentiation [109,111]. RNA sequencing analysis of adult *Tcf20* heterozygous knockout mouse brains revealed that TCF20 regulates the expression of hundreds of genes, with both upregulated and downregulated targets, suggesting that TCF20 can function as both an activator and a repressor [41]. Similar to RAI1, TCF20 binds to the promoter of *Bdnf* and regulates its expression in the mouse brain [41]. Another important downstream gene target of TCF20 is the thymine-DNA glycosylase (*Tdg*), a gene vital for DNA repair and proper neurogenesis [111]. The mechanisms and genomic locations bound by TCF20 remain unknown. Further, given that TCF20 contains an AT-hook DNA-binding domain and a histone-associating ePHD domain, the specific roles of these domains in modulating TCF20’s chromatin binding remain to be clarified.

#### 3.1.4. PHF14 and HMG20A

PHF14 is a chromatin-associated protein that regulates gene expression. It contains a tandem PHD domain (PHD1-Znk-PHD2), also referred to as its PZP domain, that binds to unmodified histone H3 (Table 3), tolerating repressive histone marks such as H3K9me3 while being repelled by active histone marks such as H3K4me3 [207]. PHF14 is recruited to promoter regions of genes implicated in proliferation, such as *Pdgfrα* [208]. Once bound, PHF14 may function as a repressor within a larger protein complex with other nuclear factors, such as the TCF20/PHF14 complex. Additionally, PHF14 enhances DNA methylation of the *SMAD7* gene by binding to unmethylated CpGs to promote TGF-β-driven metastasis in human lung adenocarcinoma [209]. This finding highlights PHF14’s role in epigenetic regulation beyond histone interaction.

HMG20A contains an intrinsically disordered region (IDR), a high mobility group domain (HMG), and a coiled-coil leucine zipper domain (CC) (Figure 1B) [210,211]. HMG20A binds to promoters of genes important for neural differentiation, such as *Synapsin*, through its HMG-DNA binding domain [52]. Although it has not been identified whether HMG20A directly binds to the epigenome, it modulates gene expression and cellular pathways by its interaction with epigenetic players, such as the histone methyltransferase MLL [52]. Whether HMG20A has other functions outside of its promoter-binding activity remains to be explored.

**Table 3:** This table summarizes the epigenetic motifs associated with each of the components of the MeCP2-interacting TCF20/PHF14 protein complex. The direct signatures that each component recognizes are shown.

Gene	Epigenetic Regulation	References
MeCP2	mCpG mCH mCAC H3K27me3 H2A H2B H3 H4	[186,187,188,189,190]
RAI1	H3K4me2 H3K4me3	[46,212]
TCF20	Unknown	NA
PHF14	Unmodified H3 CpG	[207,209]
HMG20A	Unknown	NA

NA = Not applicable.

#### 3.1.5. The Molecular Function of TCF20/PHF14 Complex

How do these transcriptional regulators interact with each other in functional chromatin complexes? Several biochemical studies have mapped the interaction domains of MeCP2, RAI1, TCF20, PHF14, and HMG20A (Figure 1B). MeCP2 interacts with TCF20 through its MBD-intervening domain (MBD-ID), while the PZP domain of PHF14 interacts with MeCP2’s MBD-ID and TCF20’s C-terminal ePHD domain, respectively, facilitating a network of connections that stabilize the complex [41]. HMG20A’s coiled-coil leucine domain interacts with PHF14 [41,210] and TCF20 interacts with RAI1 through zinc–finger interactions of their PHD domains [100], reinforcing this network’s structural integrity. It remains to be explored whether the RAI1 and HMG20A components of the TCF20/PHF14 complex bind directly to each other. In addition, determining the specific protein domain(s) of TCF20 that interact with MeCP2’s MBD-ID, as well as the domain(s) of PHF14 that interact with HMG20A’s CC domain, would help further complete the TCF20/PHF14 protein interaction map. Importantly, several *MECP2* and *PHF14* missense variants identified in NDD patients have been shown to disrupt the interaction network within this complex, highlighting the critical role of a functional TCF20/PHF14 complex in normal brain development [41]. In addition, it remains to be determined whether the pathogenic variants K1710R and H1909Y in the MeCP2/PHF14-interacting ePHD domain of TCF20 (Table 2) affect these protein–protein interactions. Ultimately, understanding how these components interact with each other and with chromatin can provide valuable insights into how NDD-related disorders arise, particularly due to haploinsufficiency or mutations affecting one or more parts of this protein complex.

The interaction network between the protein components of the TCF20/PHF14 complex implies the regulation of shared downstream gene targets (Figure 1A). As an example, MeCP2, TCF20, and RAI1 all regulate the key neuronal gene *Bdnf*, which is associated with synaptic plasticity and development [41,46,203], thus providing further insight into this complex’s role in mediating shared downstream gene targets related to NDDs. Chromatin binding data from various studies has independently shown that MeCP2, TCF20, and RAI1 each bind to the *Bdnf* promoter, though it is still unclear whether they bind as a complex or independently. The current hypothesis is that MeCP2 binds to chromatin and recruits PHF14 and TCF20 to co-regulate *Bdnf* and other downstream genes [41]. Further investigation into PHF14 and TCF20’s DNA binding patterns, especially in the absence or overabundance of MeCP2, could clarify this relationship. Additionally, given the significant overlap in downstream genes regulated by TCF20 and MeCP2 [41], it would be critical to compare their global chromatin binding patterns to elucidate the mechanisms by which these regulators synergistically control gene expression.

In addition to its role in neurodevelopment, PHF14 also forms part of another subcomplex that includes KMT2A, PHF5A, HMG20A, and RAI1, a subcomplex that epigenetically regulates pancreatic cancer stem cells through KMT2A’s methyltransferase activity [212]. This finding highlights the versatility of PHF14, HMG20A, and RAI in regulating cell identity and differentiation across various contexts, which may reveal similar mechanisms underlying their function in neuronal development.

### 3.2. Developmental Function

The expression patterns of *MECP2*, *RAI1*, *TCF20*, *PHF14*, and *HMG20A* from the human protein atlas [213] indicate that these genes are widely expressed throughout the human brain. In mice, *Mecp2* is highly expressed across the brain, with notable enrichment in most neurons [186,214,215]. Studies of *Rai1* in mice have shown its expression in both the developing and adult central nervous system, particularly in the cerebellum, the paraventricular nucleus, and the dentate gyrus [46,102,216]. *Tcf20* is broadly expressed in both the developing and adult mouse brain and displays colocalization with MeCP2 in neurons [41,111,217,218]. *Phf14* expression levels are also mapped in the adult mouse brain, with expression found in multiple brain cell types, including neurons, astrocytes, and endothelial cells [41]. *Hmg20a* is widely expressed in the developing brain, particularly in the cortex [52]. In adults, *Hmg20a* is highly expressed in the dentate gyrus of the hippocampus and in astrocytes [218,219]. Overall, the similarities and differences in the expression patterns of these genes underscore their tissue- and cell-specific roles in brain development.

The components of the MeCP2-interacting TCF20/PHF14 complex exhibit shared and distinct temporal roles during brain development. MeCP2’s expression levels in the brain start low and increase over time through postnatal development, reaching its highest levels in postmitotic neurons [214,220]. Studies using conditional mouse models show that turning off *Mecp2* in juvenile and adult mice leads to RTT-like phenotypes and triggers global transcriptional and chromatin dysregulation, with molecular deficits preceding phenotypic deficits [221,222]. Conversely, and more clinically relevant, restoring *Mecp2* expression can reverse neurological symptoms [223]. These findings, along with MeCP2’s temporal expression pattern, suggest that MeCP2 is essential for maintaining neuronal function rather than aiding early brain development and that neurological symptoms of RTT are reversible.

For *Rai1*, similar mouse studies have investigated temporal mechanisms to determine whether there is a specific developmental window during which *Rai1* is required. *Rai1’s* expression increases from prenatal development and persists into adulthood [46]. Other studies identified that restoring *Rai1* gene dosage early in development can partially rescue the transcriptional and behavioral deficits associated with *Rai1* haploinsufficiency later in life [216,224]. Interestingly, deletion of *Rai1* during adolescence or adulthood in mice leads to normal neurobehavior but induces adult-onset obesity along with reduced *Bdnf* expression [216]. Results from these mouse studies suggest that RAI1 functions in both neurodevelopment and neuronal maintenance.

Like *Mecp2* and *Rai1*, *Tcf20* mRNA levels increase during pre- and postnatal development and plateau in adulthood, suggesting that TCF20 may also play a role beyond early neurodevelopment [41]. Interestingly, TCF20 is highly expressed in the prenatal mouse brain and is critical for cortical neurogenesis [111]. However, it is still unknown whether TCF20 is necessary during postnatal development and adulthood. Given that *TCF20* and *RAI1* are homologs [100], TCF20—like RAI1—may have roles in both pre- and postnatal brain development. Thus, it would be essential to pinpoint the critical time window of TCF20’s function during development and to determine whether modulating its expression and function could provide therapeutic potential during the postnatal stage.

### 3.3. Neuronal Function

Neuronal activity drives synaptic plasticity, information processing, and memory formation in the brain. Synaptic transmission initiates transcriptional events that induce activity-dependent immediate early genes, such as *Fos*, *Egr*, *Arc*, and *Bdnf* [225], which mediate neuronal responses to stimuli, encoding experiences into memory and shaping behavior [226]. Dysregulation of activity-dependent signaling contributes significantly to NDDs [227].

The TCF20/PHF14 complex is highly expressed in neurons beyond development, suggesting a role in maintaining proper neuronal network function and activity. Disruptions in related proteins, such as MeCP2 or RAI1, have been linked to an excitatory and inhibitory imbalance as well as increased neuronal excitability, potentially contributing to specific neurological and behavioral symptoms [228,229]. These findings indicate that the TCF20/PHF14 complex may play a key role in neuronal network activity by regulating the activity-dependent transcriptome during stimulation. Consequently, the activity-dependent signaling pathway involving the TCF20/PHF14 complex may serve as a convergence point for understanding NDD pathogenesis and developing targeted therapies.

Of note, phosphorylation of MeCP2 has been shown to affect its transcriptional regulatory activities. For example, membrane depolarization induces phosphorylation of MeCP2 at S421 by CaMKII, leading to the subsequent activation of genes including *Bdnf* [197,230,231]. Furthermore, neuronal activity induced by KCl in vitro or kainic acid in vivo boosts the expression of many activity-dependent genes in mouse neurons with *Mecp2* deficiency relative to WT neurons [228]. Similarly, RAI1 also plays a crucial role in activity-dependent transcription, modulating gene expression by detaching from chromatin in response to neural activity changes and influencing synaptic scaling properties [232]. These findings indicate that both MeCP2 and RAI1 are responsive to neuronal activity and modulate downstream transcriptional pathways. While it remains unclear whether TCF20 shares similar activity-dependent roles, its common regulation of *Bdnf* suggests that it may participate in overlapping molecular pathways to modulate neuronal responses to activity.

While previous studies have relied on artificial activation methods, it is essential to study chromatin regulators within the context of physiological behavior in live animals to understand how these processes lead to behavioral abnormalities in NDDs. Notably, a study on early-life exercise in transgenic mice has revealed that MeCP2 and TCF20 function as upstream regulators of epigenetic and transcriptional changes in response to behavioral stimuli [233]. Further studies are needed to explore the activity-dependent roles of the TCF20/PHF14 complex components under physiological conditions in animal models.

## 4. Towards Therapeutics

The knowledge accumulated about protein complexes tied to NDDs, such as the TCF20/PHF14 complex, invites the opportunity for therapeutic efforts for preventing or minimizing the adverse outcomes of severe NDDs. While gene therapy has emerged as a promising approach, over the past decade, other strategies have also been developed. These strategies have been applied in clinical and preclinical studies of NDDs, such as RTT, with potential applicability to other NDDs. These strategies are described below.

### 4.1. Gene Therapies

Recent advances in CRISPR-Cas9 and prime-editing technologies have made gene therapy an increasingly viable strategy, as discussed in several review articles [234,235,236]. Remarkably, a recent study demonstrated that an RNA editing strategy could rescue gene duplication in a mouse model of MDS and nonhuman primates, providing a potential strategy for treating *MECP2*-related and other dosage-sensitive diseases [237]. However, significant challenges remain. One major challenge in applying these gene-editing technologies to NDDs is achieving efficient and uniform delivery of adeno-associated virus (AAV) vectors across the human brain. Another critical hurdle is identifying the optimal therapeutic developmental time window, which, as discussed in Section 3.2, can vary across different NDDs. Other obstacles include issues with chromatin accessibility, potential off-target effects, and high costs, which will need to be addressed.

### 4.2. Antisense Oligonucleotides (ASOs)

One promising approach involves targeting gene dosage through antisense oligonucleotides (ASOs) for monogenic NDDs. ASOs are short oligonucleotides that can bind to RNA in a target-specific manner and ultimately modify protein expressions. ASOs can reduce target RNA transcript levels, thereby limiting the production of toxic proteins, or they can modify transcript sequences through splice modulation to restore encoded protein function. Both approaches have been shown in animal models to improve symptoms and/or delay the onset of certain NDDs [238,239]. For instance, ASOs have shown efficacy of reducing protein levels in mouse models of Dravet syndrome and MDS, consequently reducing or reversing related phenotypes and extending survival [240,241,242]. Additionally, splice-switching ASOs can be used to restore functional protein levels in mouse models of Dravet syndrome and brain calcification [243,244]. To date, the Food and Drug Administration (FDA) has approved more than 13 ASOs for clinical use, with several others currently undergoing clinical trials [239,245]. This progress highlights the potential of ASOs as a therapeutic strategy for NDDs caused by mutations in genes including *MECP2*, *TCF20*, or *RAI1*.

### 4.3. New Drug Discovery

Since DNA binding is crucial for the function of transcription regulators, identifying small molecules that modulate DNA binding of mutant proteins may restore protein function and alleviate associated clinical symptoms. However, quantitative measurement of this property, especially in live neurons under physiological conditions, remains challenging. Recently, single-particle tracking (SPT) has enabled live-cell imaging of chromatin proteins using fluorescent probes, providing insights into their nuclear dynamics associated with NDD pathogenesis [246,247]. This technique has been used to study mutations in MeCP2 and quantify its chromatin dynamics, revealing that certain variants affect MeCP2’s chromatin association [184,248]. Thus, the combination of this live-cell imaging tool with newly established cellular models of NDD provides a platform to directly screen and evaluate new therapeutic interventions that can normalize the DNA-binding capacity of pathogenic MeCP2 or other chromatin-associated proteins [249].

### 4.4. Electrical or Behavioral Stimulation

Beyond specific gene-targeting strategies, therapies that modulate activity-dependent brain signaling may also offer potential benefits. Deep brain stimulation (DBS) and repetitive behavioral training are other possible therapeutic approaches for NDDs. DBS involves the use of electrodes to send electric pulses to the brain for modulating neuronal circuits, which have been shown to improve symptoms of ASD, obsessive–compulsive disorder, and Tourette syndrome [250,251,252]. Preclinical in vivo studies have demonstrated that DBS restores hippocampal memory in RTT mouse models [253,254,255,256], underscoring its translational potential for treating RTT and other NDDs.

Another option is behavioral therapy, such as applied behavior analysis (ABA), which is non-invasive and can be beneficial for individuals with ASDs, with potential applicability to other NDDs. Recent studies on RTT mouse models have shown that presymptomatic repetitive training can improve RTT-like phenotypes later in life and delay symptom onset [257,258]. It would be exciting to explore whether early behavioral training could similarly ameliorate neurobehavioral abnormalities in RTT patients. Moreover, given the involvement of other components in the TCF20/PHF14 complex—such as TCF20 and RAI1—in activity-dependent signaling pathways, modulating neural activity could similarly address LoF mutations in these genes, potentially rescuing associated behavioral abnormalities. This approach represents a promising avenue for expanding therapeutic strategies in NDDs.

## 5. Conclusions

Dysregulation of chromatin and transcriptional regulation are increasingly recognized as central factors in the pathogenesis of NDDs. Given the heterogeneity of NDDs, it remains challenging to address the underlying causes comprehensively. The MeCP2-interacting TCF20/PHF14 complex offers a focused model for understanding a subset of these disorders, as variants in its components are associated with NDDs displaying overlapping phenotypes. This overlap highlights the convergence of NDD-associated genes on shared molecular pathways and emphasizes the importance of studying these complexes to uncover fundamental mechanisms. Technological advancements such as gene editing and single-molecule imaging promise new opportunities to elucidate the molecular and neurological functions of this complex. Future research leveraging these innovative approaches could not only enhance our understanding of the TCF20/PHF14 complex but also shed light on other chromatin-regulating complexes implicated in NDDs. These findings may ultimately inform the design of targeted therapeutics, offering hope for more effective treatments and better outcomes for individuals affected by these challenging conditions.
